# Self-Organization of Enzyme-Catalyzed Reactions Studied by the Maximum Entropy Production Principle

**DOI:** 10.3390/ijms24108734

**Published:** 2023-05-13

**Authors:** Andrej Dobovišek, Marko Vitas, Tina Blaževič, Rene Markovič, Marko Marhl, Aleš Fajmut

**Affiliations:** 1 Faculty of Natural Sciences and Mathematics, University of Maribor, Koroška Cesta 160, 2000 Maribor, Slovenia; 2 Faculty of Medicine, University of Maribor, Taborska Ulica 8, 2000 Maribor, Slovenia; 3 Laze pri Borovnici 38, 1353 Borovnica, Slovenia; 4 Faculty of Electrical Engineering and Computer Science, University of Maribor, Koroška Cesta 46, 2000 Maribor, Slovenia; 5 Faculty of Education, University of Maribor, Koroška Cesta 160, 2000 Maribor, Slovenia; 6 Faculty of Health Sciences, University of Maribor, Žitna Ulica 15, 2000 Maribor, Slovenia

**Keywords:** enzymes, kinetic data analysis, steady state, self-organization, maximum entropy production

## Abstract

The self-organization of open reaction systems is closely related to specific mechanisms that allow the export of internally generated entropy from systems to their environment. According to the second law of thermodynamics, systems with effective entropy export to the environment are better internally organized. Therefore, they are in thermodynamic states with low entropy. In this context, we study how self-organization in enzymatic reactions depends on their kinetic reaction mechanisms. Enzymatic reactions in an open system are considered to operate in a non-equilibrium steady state, which is achieved by satisfying the principle of maximum entropy production (MEPP). The latter is a general theoretical framework for our theoretical analysis. Detailed theoretical studies and comparisons of the linear irreversible kinetic schemes of an enzyme reaction in two and three states are performed. In both cases, in the optimal and statistically most probable thermodynamic steady state, a diffusion-limited flux is predicted by MEPP. Several thermodynamic quantities and enzymatic kinetic parameters, such as the entropy production rate, the Shannon information entropy, reaction stability, sensitivity, and specificity constants, are predicted. Our results show that the optimal enzyme performance may strongly depend on the number of reaction steps when linear reaction mechanisms are considered. Simple reaction mechanisms with a smaller number of intermediate reaction steps could be better organized internally and could allow fast and stable catalysis. These could be features of the evolutionary mechanisms of highly specialized enzymes.

## 1. Introduction

Enzymatic reactions are the most common biochemical reactions in living cells and are essential for almost all cellular processes. The structures, functions, and kinetic properties of modern enzymes have changed considerably during evolution. Since biological evolution is widely regarded as an optimization process, the evolution of enzymes could be explained as the result of evolutionary optimization [1,2,3,4,5]. In recent decades, various optimization principles have been proposed to explain the kinetic properties of modern enzymes [1,2,3,4,5,6,7,8,9,10,11,12,13,14,15,16,17], but a general agreement on this topic has not yet been reached among researchers.

From a thermodynamic point of view, according to the second law of thermodynamics, the change in entropy dictates the direction of all spontaneous processes in nature. The general form of the second law of thermodynamics is: $dS=d_{i}S+d_{e}S$, where *dS* is the total net change in entropy of the system, $d_{i}S$ is the entropy generated by irreversible processes within the system, and $d_{e}S$ is the entropy exported from the system to its environment. While $d_{i}S$ can only be positive, $d_{e}S$ can be either zero or negative. For a closed system where $d_{e}S=0$, the total net entropy change always increases or remains constant. Hence, $dS=d_{i}S\geq 0$. In this case, the system spontaneously approaches the equilibrium state with maximal entropy. However, if the system is open and can exchange heat and matter with its environment, it can avoid the equilibrium state by exporting internally generated entropy to its environment [18]. If such a mechanism allows efficient, energetic decomposition of incoming metabolites (energy dissipation) and efficient export of internally generated entropy to the environment, the system can make a spontaneous transition from a state of higher to a state of lower entropy and thus spontaneously become better organized [18,19]. It follows from thermodynamic laws that any system characterized by an irreversible flow of entropy will automatically self-organize [18,19]. Such spontaneously self-organizing processes are statistically highly improbable (though possible) in closed thermodynamic systems but highly probable in open systems. Open systems can persist longer in ordered states with lower entropy because, according to the second law of thermodynamics, a nontrivial and stable steady state with *dS* = 0 for $d_{e}S=-d_{i}S\leq 0$ can occur. Hence, in open systems, highly ordered systems under non-equilibrium conditions can spontaneously set up, producing entropy and releasing it to the environment [18]. Biological systems are considered as such.

An enzyme-catalyzed reaction possesses a specific reaction mechanism in which the catalytic site of the enzyme attracts substrate molecules from the environment, converts them into a product (dissipates energy), and then releases them back into the environment. These mechanisms are represented by the kinetic reaction schemes and described by the corresponding kinetic models. Different kinetic mechanisms of enzyme reactions have evolved during biological evolution in response to different environmental and metabolic conditions [20]. Enzymatic reactions in living cells are supposed to take place in open reaction systems under non-equilibrium conditions in (more or less) stable quasi-steady states. Such thermodynamic states are generally characterized by slowly changing thermodynamic forces and fluxes, resulting in continuous entropy production and dissipation of free energy. Their properties could be explained by irreversible steady-state thermodynamics rather than reversible equilibrium thermodynamics. Several researchers [21,22,23,24] have identified the principle of maximum entropy production (MEPP), also known as the MEP conjecture, as a basic assumption for the thermodynamic analysis of non-equilibrium steady states. According to MEPP, an open thermodynamic system spontaneously evolves towards the steady state characterized by the maximal entropy production rate. The system reaches this steady state spontaneously because it is statistically most probable [23,24,25,26]. There is general agreement that MEPP is a suitable optimization approach for the analysis of stationary near-equilibrium processes in which a linear force-flow relationship holds [27]. In the analysis of non-equilibrium stationary processes, MEPP is adopted as a working hypothesis [27]. Several published papers show that enzymes and metabolic networks operate according to MEPP [10,11,12,13,14,15,16,17,28,29]. Based on this knowledge, it could be suggested that MEPP could be one of the most important thermodynamic principles explaining the kinetic properties of modern enzymes.

MEPP is often used for analyzing various open non-equilibrium systems in physics, chemistry, and biology [22]. According to MEPP, the entropy production of the system is maximized with respect to one or more selected system parameters [30]. In this way, parameter values that account for the optimal thermodynamic performance of the system in a steady state are calculated. Different theoretical formulations of MEPP exist in the literature [22]. In this study, the entropy production of the system is maximized by searching for maximal overall reaction flux at fixed thermodynamic force. MEPP is also closely related to the principle of maximal Shannon information entropy (MaxEnt). Namely, the non-equilibrium steady state of an open system characterized by maximal entropy production rate should, at the same time, also be characterized by the maximal value of the Shannon information entropy [23,24,25,26]. This means that the steady state of maximal entropy production (MEP) is also statistically the most probable steady state of the system. On the other hand, it has been found that MEPP not only governs the evolution of non-equilibrium physical and chemical processes but is also closely related to different hypotheses from evolutionary biology [31].

This work uses MEPP as a theoretical thermodynamic framework to study the self-organization of linear irreversible enzyme reactions. The goal is to gain new insights into how the kinetic reaction mechanism of a linear enzyme reaction affects quantities such as the entropy production rate, the catalytic efficiency of a reaction, the Shannon information entropy of a reaction, the reaction sensitivity to external perturbations, the reaction stability, and standard kinetic parameters such as the Michaelis–Menten constant, the catalytic constant, and the specificity constant. To this end, two- and three-state irreversible kinetic schemes of an enzyme reaction are investigated and compared in detail. For both reaction schemes, MEPP predicts that the reaction flux of an enzyme reaction in its most probable thermodynamic steady state should be controlled by the diffusion of the reactants. Optimal solutions for the Michaelis–Menten constant, the catalytic constant, and the specificity constant are derived analytically. These solutions resemble in all aspects the kinetic criteria for diffusion-limited or catalytically optimal (perfect) enzymes. The results of our study suggest that reaction mechanisms may be essential for the optimization of enzyme performance and the self-organization of biochemical reactions. Theoretical solutions show that all thermodynamic and kinetic properties of enzymatic reactions strongly depend on the number of intermediate reaction steps or, equivalently, on the number of intermediate enzymatic functional states within the whole reaction mechanism. In this context, reaction mechanisms with a smaller number of intermediate functional states are, by default, better internally organized and characterized by lower Shannon information entropy. This is reflected in a higher entropy production rate, better catalytic performance, and higher reaction stability and response to external perturbations.

## 2. Results and Discussion

### 2.1. Optimal Enzyme State and Diffusion-Limited Enzymes

**Figure 1:**
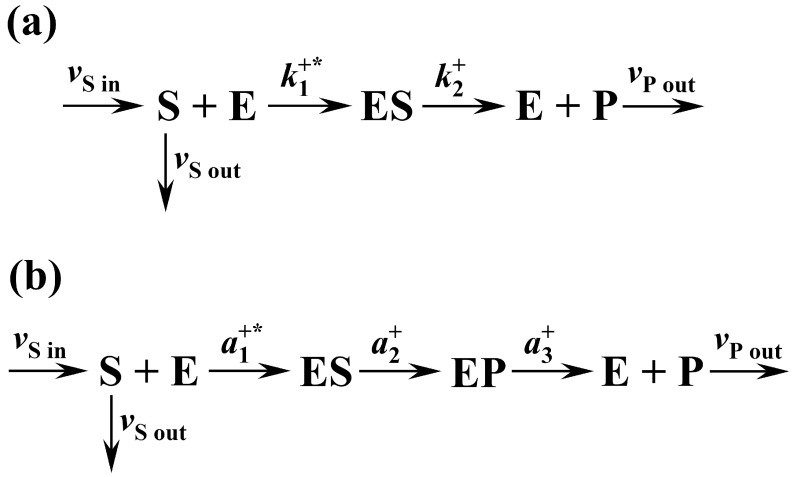
Kinetic schemes of an enzyme reaction considered for thermodynamic analysis with MEP principle. (**a**) Kinetic scheme of the irreversible two-state enzyme reaction. E and ES are enzyme functional states of a free enzyme and an enzyme in complex with the substrate, respectively. Transitions between the enzyme’s functional states are described by the enzyme rate constants: $k_{1}^{+*}$—second-order (diffusion) forward rate constant and $k_{2}^{+}—$first-order forward rate constant. (**b**) Kinetic scheme of the irreversible three-state enzyme reaction. E, ES, and EP are the functional states of a free enzyme, an enzyme in complex with a substrate, and an enzyme in complex with a product, respectively. Transitions between enzyme states are described by the enzyme rate constants, which are designated as: $a_{1}^{+*}$—second-order (diffusion) forward rate constant, and $a_{2}^{+},\;a_{3}^{+}$—first-order forward rate constants. In both cases, open reaction systems are considered in a non-equilibrium steady state. The symbols S and P designate a substrate and a product, respectively. The symbols $v_{S\;\mathrm{in}}$, $v_{S\;\mathrm{out}}$, and $v_{P\;\mathrm{out}}$ designate the external incoming and outgoing fluxes of metabolites, which are: $v_{S\;\mathrm{in}}$—incoming flux of a substrate, $v_{S\;\mathrm{out}}$—outgoing flux of a substrate, and $v_{P\;\mathrm{out}}$—outgoing flux of a product.

Optimal values of the enzyme rate constants, corresponding to the maximal rate of entropy production in an irreversible two-state kinetic scheme, shown in Figure 1a, can be easily analytically derived and calculated by using Equation (28). Since the numerator of Equation (28) is constant, maximal reaction flux is achieved when the denominator of this equation is minimal. Demand that the first derivative of the denominator in Equation (28) with respect to $k_{2}^{+}$ equals zero yields $k_{2\;\mathrm{op}}^{+}=\sqrt{K^{+}S}$. By considering $K^{+}=k_{1}^{+*}k_{2}^{+}=k_{1\;\mathrm{op}}^{+*}k_{2\;\mathrm{op}}^{+}$ we directly obtain $k_{1\;\mathrm{op}}^{+*}\left[S\right]=k_{2\;\mathrm{op}}^{+}$, which is, in our model, a global optimality condition for establishing a steady state with a maximal entropy production rate. In the above condition, the rate constant $k_{2}^{+}$ describes the pure enzymatic catalytic step ES→E+P. On the other hand, the product $k_{1\;}^{+*}\left[S\right]$ is known as the pseudo-first-order rate constant, which describes the transition S+E→ES and depends on the second-order diffusion rate constant $k_{1\;}^{+*}$ as well as on the substrate concentration [S]. The parameter $k_{1}^{+*}$ describes the diffusion of substrate molecules to the enzyme’s active site and is a subject of optimization in our model. [S] is the enzyme’s steady-state substrate concentration. In our further analysis, we demonstrate that condition $k_{1\;\mathrm{op}}^{+*}\left[S\right]=k_{2\;\mathrm{op}}^{+}$ globally affects practically all aspects of the enzyme thermodynamics and kinetics and implies that the reaction flux of an enzyme reaction is under the condition of maximal entropy production rate controlled only by diffusion. Even more, it will be demonstrated that this is also statistically the most probable steady state of a reaction. We have also shown that the above condition predicted by MEPP represents a reasonable theoretical basis for the thermodynamic explanation of the already-known features of diffusion-controlled enzymes that operate in a catalytically optimal manner. Such enzymes exist in nature, and their features are well described in terms of Michaelis–Menten $\left(K_{M}\right)$ and specificity $\left(k_{\mathrm{cat}}/K_{M}\right)$ constants.

The condition $k_{1\;\mathrm{op}}^{+*}\left[S\right]=k_{2\;\mathrm{op}}^{+}$ dictates that the steady state with a maximal rate in the entropy production is established when all forward rate constants are equal. It is generally known that these parameters are different when considering individual enzymes. The internal enzymatic reaction steps described by $k_{2}^{+}$ could generally be much faster than the diffusion of substrates to the enzyme’s active site, as described by $k_{1}^{+*}$. However, according to the above optimality condition, very fast internal enzymatic molecular transitions do not improve the enzyme’s catalysis performance. Diffusion to the active site is a rate-limiting step for the entire reaction and dictates the upper value of the overall reaction flux in a steady state. It follows directly from our theoretical model that the maximal rate of entropy production and, thus, maximal reaction rate are achieved if internal enzymatic reaction steps are as fast as the substrate’s diffusion to the enzyme catalytic site.

Replacement of the optimal forward rate constant $k_{\mathrm{op}}^{+}$ $\left(k_{\mathrm{op}}^{+}=\sqrt{K^{+}S}\right)$ with $k_{\mathrm{op}}^{+}=k_{1\;\mathrm{op}}^{+*}\left[S\right]=k_{2\;\mathrm{op}}^{+}$ in Equations (19) and (20), accounting for the state with maximal entropy production rate, results in equal steady-state concentrations $\left[E\right]=\left[ES\right]={\left[E\right]}_{\mathrm{tot}}/2$. According to Equation (5), this results in a uniform probability distribution of the enzyme’s functional states $p_{1}=p_{2}=0.5$, and, according to Equation (4), in the maximal Shannon information entropy H=ln(2). This result highlights that the steady state with the maximal rate in entropy production is statistically also the most probable steady-state of the reaction. The same result can be obtained by considering $k_{\mathrm{op}}^{+}=k_{1\;\mathrm{op}}^{+*}\left[S\right]=k_{2\;\mathrm{op}}^{+}$ in Equation (29).

**Figure 2:**
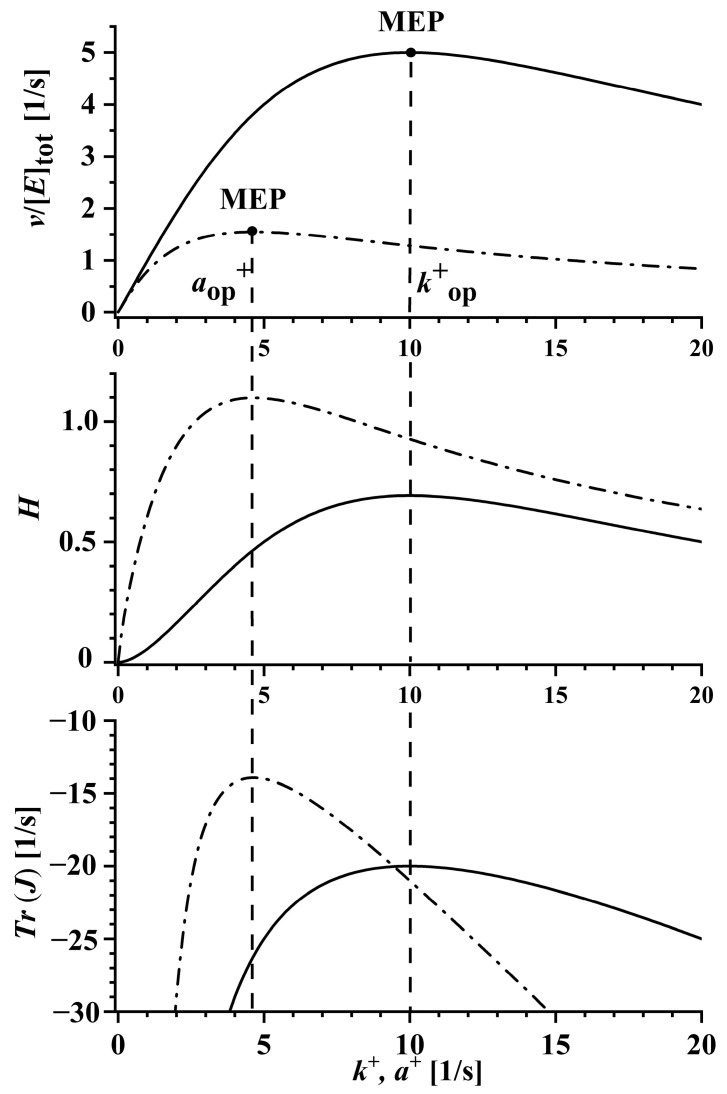
Thermodynamic optimization of the two- (solid lines) and the three-state (dash-dot lines) irreversible enzyme reaction: steady-state reaction flux normalized with the total enzyme concentration ($v\;/{\left[E\right]}_{\mathrm{tot}})$ (upper panel), Shannon information entropy (H) (middle panel), and the trace of the Jacobian matrix (Tr(J)) (lower panel) in dependence on the enzyme forward rate constants $k^{+}$ or $a^{+}$ for the two- and the three-state kinetic schemes, respectively. The optimal value of the rate constants $k_{\mathrm{op}}^{+}$ and $a_{\mathrm{op}}^{+}$ corresponds with the maximal reaction flux, the maximal Shannon information entropy, and the maximal trace of the Jacobian matrix. Calculations are performed for the following values of the model parameters: [S]=1 μM, $K^{+}=100\;{\mu M}^{-1}s^{-2}$ (for the two-state kinetic scheme) and [S]=1 μM, $K^{+}=100\;{\mu M}^{-1}s^{-3}$ (for the three-state kinetic scheme).

A closer inspection of the reaction stability, described by the trace of the Jacobian matrix (Equation (30)), shows that Tr(J) is equal (except for the minus sign) to the denominator of Equation (28) for the reaction flux. It thus possesses a unique maximal value $\mathrm{Tr}\left(J\right)=-2k_{\mathrm{op}}^{+}$ for the same optimal rate constant $k_{\mathrm{op}}^{+}$ and the same condition $k_{1\;\mathrm{op}}^{+*}\left[S\right]=k_{2\;\mathrm{op}}^{+}$ as the rate of entropy production and the Shannon information entropy; this result is graphically presented in Figure 2 for the two- and three-state kinetic schemes, solid and dash-dot lines, respectively. As can be seen from the bottom panel of Figure 2, the largest values of Tr(J) (i.e., the least negative among all possible states) for both reaction schemes are consistent with the statistically most probable steady states predicted with MEPP and Shannon entropy. This result suggests that the statistically most probable state is the least stable and, therefore, the most flexible among all possible steady states.

The optimality condition $k_{1\;\mathrm{op}}^{+*}\left[S\right]=k_{2\;\mathrm{op}}^{+}$ allows the prediction of the optimal Michaelis–Menten constant for the two-state kinetic scheme. According to Equation (24) and the condition $k_{1\;\mathrm{op}}^{+*}\left[S\right]=k_{2\;\mathrm{op}}^{+}$, the optimal $K_{M}$ for the two-state reaction scheme is $K_{M}=\left[S\right]$. In general, for enzyme pathways operating at total capacity, optimal $K_{M}$ values for many enzymes are equal to or slightly higher than physiological substrate concentrations [6,8,32,33]. Accordingly, we obtained $K_{M}=\left[S\right]$ for the diffusion-limited enzymes operating at the maximal reaction flux predicted by MEPP. We can further derive the optimal value of the specificity constant, which is defined as the ratio $k_{\mathrm{cat}}/K_{M}$. That is, by considering Equations (23) and (24), and the condition $k_{1\;\mathrm{op}}^{+*}\left[S\right]=k_{2\;\mathrm{op}}^{+}$, the optimal specificity constant for the two-state kinetic scheme is equal to the optimal second-order (diffusion) rate constant $k_{\mathrm{cat}}/K_{M}=k_{1\;\mathrm{op}}^{+*}$. If the specificity constant $k_{\mathrm{cat}}/K_{M}$ is similar in magnitude to the diffusion rate constant $k_{1}^{+*}$, then according to [6,8,32,33], the enzyme is considered to be catalytically optimal. As seen here, the condition $k_{1\;\mathrm{op}}^{+*}\left[S\right]=k_{2\;\mathrm{op}}^{+}$, predicted by MEPP, is global and implies that the statistically most probable steady states of enzyme reactions are characterized by a maximal diffusion-limited reaction flux, which is a characteristic of the catalytically optimal or diffusion-limited enzymes. As explicitly described here for the two-state irreversible kinetic scheme, the same analysis is also performed for the three-state kinetic scheme shown in Figure 1b. Modeling and analysis for the latter case are detailed in Appendix A. In the following subsection, we present and discuss a detailed comparison of the results obtained for both cases.

### 2.2. Broader Comparison of the Optimized Two- and Three-State Irreversible Kinetic Schemes

Enzymatic reactions investigated in experiments are observed as a series of multiple, more or less stable, enzymatic functional states. The kinetic schemes of enzyme reactions proposed by experimentalists include primarily only the most stable or most representative states [34]. With that in mind, the two- and three-state kinetic schemes in Figure 1a,b are here viewed as two isoenzymes that catalyze the same substrate to the same product, first according to an irreversible two-state kinetic scheme (Figure 1a) and second with a corresponding three-state kinetic scheme (Figure 1b). The values of both overall reaction equilibrium constants $K^{*}$ for these two isoenzymes must be the same, since $K^{*}$ depends only on the chemical nature of the substrate and the product. We further assume identical environmental conditions, substrate and product concentrations, and the same values of the parameter $K^{+}$ for both isoenzymes. Both reactions are placed in their statistically most probable steady state with the corresponding maximal values of the Shannon information entropy and the rate of entropy production. We examine whether the two isoenzymes under the same conditions have significant kinetic and thermodynamic efficiency differences resulting from the differences in reaction mechanisms. To investigate this, we compare optimal values of the quantities $k_{\mathrm{op}}^{+}$, $v/{\left[E\right]}_{\mathrm{tot}}$, H, Tr(J), $\varepsilon_{S}^{v}$, $k_{\mathrm{cat}}$, $K_{M}$, and $k_{\mathrm{cat}}/K_{M}$ for both reactions. In calculations, we use a typical order of magnitude for the substrate concentration [S]~1 μM and the values $K^{+}=100\;{\mu M}^{-1}s^{-2}$ and $K^{+}=100\;{\mu M}^{-1}s^{-3}$ for the two- and the three-state kinetic scheme, respectively. The value $K^{+}=100\;{\mu M}^{-1}s^{-2}$ is chosen such that the predicted optimal diffusion rate constant for the two-state kinetic scheme is $k_{1}^{+*}=10^{7}{\left(\mathrm{Ms}\right)}^{-1}=10\;{\left(\mu \mathrm{Ms}\right)}^{-1}$, which is an order of magnitude lower than the maximal experimentally determined value of this parameter ($10^{8}-10^{9}\;{\left(\mathrm{Ms}\right)}^{-1}$) [4]. The latter value is the upper limit of the diffusion-controlled binding step within the enzyme reaction kinetics [4]. A detailed comparison of the results, calculated for both kinetic schemes, is presented in Table 1.

The results presented in Table 1 show that optimal enzyme performance strongly depends on the number of enzyme functional states involved in the enzyme reaction mechanism. Based on the comparison of the results shown in Table 1, we can conclude that, under identical conditions, simple reaction mechanisms with a smaller number of enzyme states, according to the model, result in faster and more efficient catalysis of a given substrate compared to the reaction mechanisms with a larger number of enzyme states. For example, the results in the first row of Table 1 show that the optimal $k_{\;\mathrm{op}}^{+}$ for the two-state kinetic scheme is higher than the corresponding kinetic constant $a_{\;\mathrm{op}}^{+}$ for the three-state kinetic scheme despite the same conditions. Thus, this result is independent of the environmental conditions and is, as in reference [35], considered an intrinsic feature of a given kinetic scheme.

The result can also be further generalized. Hence, for a linear scheme of an enzyme reaction with *n* enzyme functional states, the generalized expression for the optimal rate constant would be:

(1) $$k_{\mathrm{op}}^{+}=\sqrt[{n}]{K^{+}[S]}$$

As seen, $k_{\mathrm{op}}^{+}$ and the optimal solutions of all other quantities and parameters in Table 1, which depend on $k_{\mathrm{op}}^{+}$, decrease with increasing *n*. Accordingly, in the two-state kinetic scheme, a higher value of $k_{\mathrm{op}}^{+}$ results in the highest value of the reaction flux normalized by the total enzyme concentration (second row in Table 1). Even if the optimal kinetic rate constants for both kinetic schemes were the same, i.e., $k_{\;\mathrm{op}}^{+}=a_{\;\mathrm{op}}^{+}$ (in our model, this is possible for [S]=1 μM and $K^{+}=1$), the steady-state reaction flux in the two-state kinetic scheme would be higher as in a three-state scheme, i.e., $v\;/{\left[E\right]}_{\mathrm{tot}}=k_{\;\mathrm{op}}^{+}/2$ and $v\;/{\left[E\right]}_{\mathrm{tot}}=k_{\;\mathrm{op}}^{+}/3$, respectively. As seen from the third row of Table 1, the maximal value of the Shannon information entropy in the two-state kinetic scheme is lower than in the three-state scheme. This is consistent with the generalized form of the second law of thermodynamics described in the Introduction. In our case, the maximal Shannon information entropy calculated for the two-state kinetic scheme (H=ln(2)) is lower than that in the three-state kinetic scheme (H=ln(3)). Further comparison of the model calculations shows that the optimal value of the trace of the Jacobian matrix (Tr(J)) (the fourth row of Table 1) for the two-state scheme is lower (more negative) than that for the three-state scheme. According to several references [36,37,38,39,40], this is associated with greater reaction stability of the two-state reaction mechanism (see lower panel of Figure 2). From the model results presented in the fifth row of Table 1, it can further be seen that the optimal value of the catalytic constant $k_{\mathrm{cat}}$ (reaction flux normalized by the total enzyme concentration at very high substrate concentration) for the two-state kinetic scheme is again higher than that for the three-state kinetic scheme.

**Figure 3:**
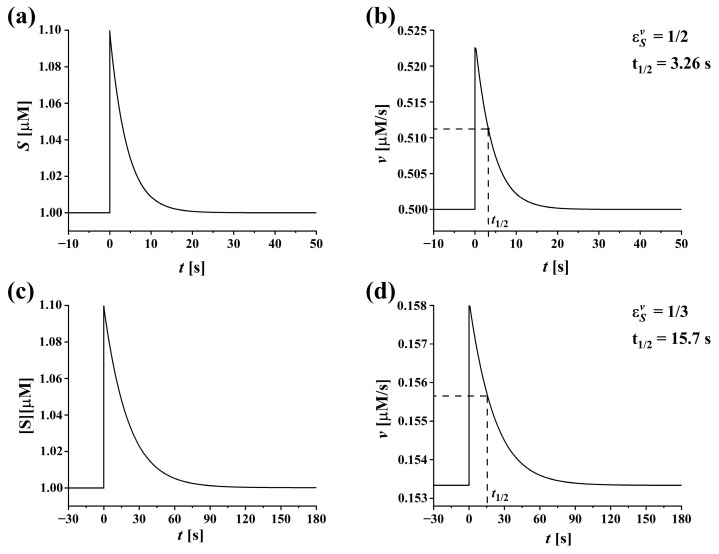
Time-responses of the reaction flux v(t) and the substrate concentration [S] (t) to a transient increase in the substrate concentration [S] above the steady-state value. (**a**,**c**) show the time-dependences of the substrate concentration [S] (t) after a transient 10 % increase above the steady-state value and its decay back to a steady state for the two- and three-state kinetic schemes, respectively. (**b**,**d**) show the corresponding response of the reaction fluxes v(t) for the two- and three-state kinetic schemes, respectively. The transient [S] (t) is simulated as follows: at *t* < 0 [S]=1 μM, at *t* = 0 [S]=1.1 μM, and at *t* > 0 [S](t) is calculated with the corresponding dynamical system for either two- (Equations (12)–(15)) or three-state (Equations (A1)–(A5)) kinetic schemes. These two dynamical systems also calculate the reaction fluxes v(t ). The total enzyme concentration used in the simulations is ${\left[E\right]}_{\mathrm{tot}}=0.1\;\mu M,$ and the values of the kinetic parameters $k_{\mathrm{op}}^{+}$ and $a_{\mathrm{op}}^{+}$ used are given in Table 1.

The results presented in the second and the sixth row of Table 1 are discussed together. The expression obtained for the optimal reaction flux normalized by the total enzyme concentration in the two-state kinetic scheme (second row of Table 1) shows that optimal flux equals one-half of the saturation flux $v\;/{\left[E\right]}_{\mathrm{tot}}=k_{\mathrm{op}}^{+}/2$. The optimal Michaelis–Menten constant ($K_{M}$) for that case (row six in Table 1) is $K_{M}=\left[S\right]$. These two results imply that optimal flux in the two-state kinetic scheme is achieved at the steady-state [S], which is also physiologically most effective. That is indeed an ideal case for achieving optimal enzyme catalysis. As can be seen from Table 1, this is not quite the same for the three-state kinetic scheme since the optimal reaction flux is equal to one-third of the saturation flux $v\;/{\left[E\right]}_{\mathrm{tot}}=a_{\mathrm{op}}^{+}/3$ and the optimal Michaelis–Menten constant ($A_{M}$) is by a factor 2 lower than the optimal physiological value of [S], i.e., $A_{M}=\left[S\right]/2$. These differences also result in different flux regulations. Enzymes operating according to the three-state kinetic scheme are saturated at lower substrate concentrations. Therefore, they may not be as sensitive (compared to the two-state case) to changes in substrate concentrations around the physiological values of [S]. That is demonstrated using numerical simulations. Figure 3 shows the response of the reaction flux with respect to the time-dependent substrate concentration for both kinetic schemes. Typical signals used in numerical simulations are shown in Figure 3a,c. In both reactions, steady states with a maximal rate of entropy production were first established at the substrate concentration [S]=1 μM. The initial steady state is in both figures presented for the negative values of time (*t* < 0). Then, at *t* = 0, a transient 10% increase in [S] is generated, followed by the time-dependent decay of [S] (*t* > 0) as predicted by the dynamical system for each kinetic scheme. The resulting time courses of the reaction fluxes are then calculated with both kinetic models. Despite the same initial substrate concentrations in the steady state and the same increase in [S] at *t* = 0, the corresponding response of reaction flux at *t* = 0 is higher for the two-state (Figure 3b) than for the three-state kinetic scheme (Figure 3d). This is a consequence of a higher elasticity coefficient $\varepsilon_{S}^{v}$ in the case of the two-state reaction scheme (see the last row in Table 1). As can be seen from Equation (27), $\varepsilon_{S}^{v}$ depends on the initial steady-state [S] and the parameter $K_{M}$. Since [S] is equal in both cases, the differences in flux responses are due to different optimal $K_{M}$ (or $A_{M}$) values for both kinetic schemes, an intrinsic property of the specific reaction mechanism [35]. As shown in Figure 3, the decay rate, which is inversely proportional to a half-decay time ($t_{1/2}$), of either variable [S] or the reaction flux v, as seen in Figure 3b,d, is also much higher in the two-state as in the three-state kinetic scheme. That is due to differences in the stabilities of the reaction systems, which is reflected in different values of Tr(J).

Finally, we discuss the results obtained for the specificity constants in row seven of Table 1. The specificity constants $\left(k_{\mathrm{cat}}/K_{M}\right),(a_{\mathrm{cat}}/A_{M})$, for the two- and three-state kinetic schemes, respectively, predicted by MEPP, are for both cases equal to the corresponding second-order diffusion rate constant $k_{\;1\;\mathrm{op}}^{+*}$ and $a_{\;1\;\mathrm{op}}^{+*}$. That is expected for diffusion-limited enzymes. Since the specificity constants depend on $k_{\mathrm{cat}}$ and $a_{\mathrm{cat}}$, which are different for the two kinetic schemes, the specificity constant also depends on the reaction mechanism. This result contrasts previous theoretical studies [4,41,42,43] where authors assumed that the second-order diffusion rate constant should or might be considered entirely independent of specific reaction mechanisms, depend solely on diffusional factors, and remain unaffected during evolutionary enzymatic development. For example, Pettersson [4] carried out a theoretical investigation on the evolution of enzyme kinetics and assumed that the second-order rate constant is a fixed parameter in the history of enzyme evolution with the highest possible value $k_{1}^{+*}=10^{9}\;{\left(\mathrm{Ms}\right)}^{-1}$, which is the upper limit for the diffusion-controlled binding step. In contrast, the results of our model show that the second-order rate constant in calculating the optimal rate constant $k_{\mathrm{op}}^{+}$ depends on the reaction mechanism. If we recall the generalized expression for the optimal rate constant of the *n*-step reaction mechanism $k_{\mathrm{op}}^{+}=\sqrt[{n}]{K^{+}\left[S\right]}$ (see Equation (1)) and take into account the well-known relation between the first- and the second-order rate constant $k_{\mathrm{op}}^{+}=k_{\mathrm{op}}^{+*}\left[S\right]$ [34], it follows that $k_{\mathrm{op}}^{+*}=\sqrt[{n}]{K^{+}\left[S\right]}/\left[S\right]$. Thus, if $K^{+}$ and [S] are considered fixed model parameters, the optimal diffusion rate constant decreases as *n* increases. Hence, both $k_{\mathrm{op}}^{+*}$ and $k_{\mathrm{cat}}/K_{M}$ depend on the intrinsic properties of a given reaction mechanism, which is a direct theoretical result of applying MEPP. This agrees with the discussion in reference [35]. Accordingly, the specificity constant for the two-state kinetic scheme is slightly higher than that calculated for the three-state scheme. However, both results are still within the same order of magnitude.

**Figure 4:**
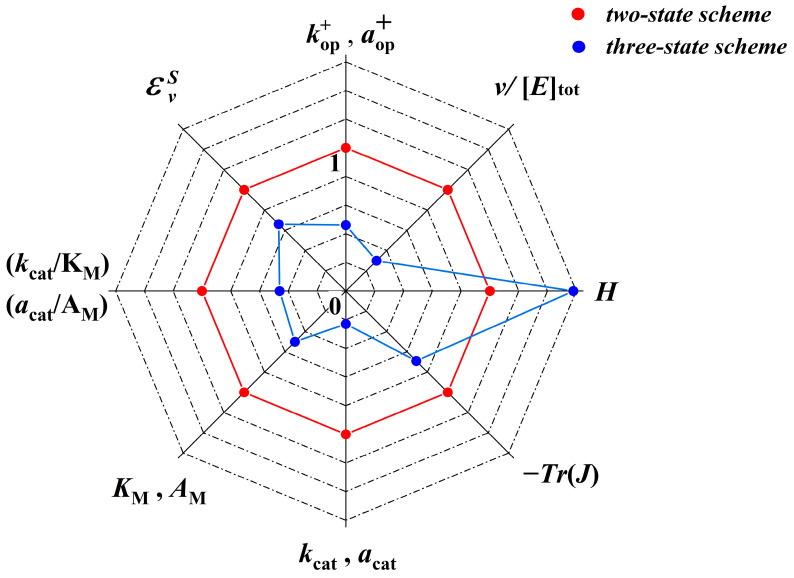
Graphical comparison of the optimal reaction parameters for the two- and three-state kinetic schemes of an enzyme reaction. The optimal values of individual parameters for the three-state scheme (blue color) are expressed as a proportion of the corresponding two-state scheme values (red color). Legend: $k_{\;\mathrm{op}}^{+},\;a_{\mathrm{op}}^{+}$—forward rate constants for the two- and three-state kinetic schemes, respectively, $v\;/{\left[E\right]}_{\mathrm{tot}}$—steady-state reaction flux normalized with the total enzyme concentration, *H*—Shannon information entropy, Tr(J)—trace of the Jacobian matrix, $k_{\mathrm{cat}}$, $a_{\mathrm{cat}}$—enzyme catalytic rate constants for the two- and three-state kinetic schemes, respectively, $K_{M}$, $A_{M}$—Michaelis–Menten constants, $k_{\mathrm{cat}}/K_{M}$, $a_{\mathrm{cat}}/A_{M}$—specificity constants, and $\varepsilon_{S}^{v}$—elasticity coefficient.

Finally, Figure 4 summarizes all predicted optimal reaction parameter values and enables their comparison for the two- and three-state kinetic schemes in a single graphical representation. The optimal values of each parameter for the three-state scheme are expressed as a proportion of the corresponding two-state scheme values.

**Figure 5:**
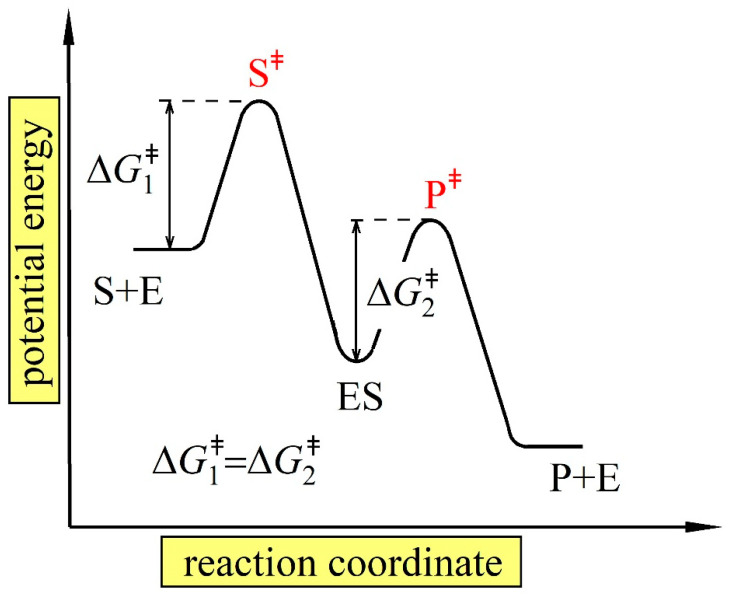
Schematical representation of the potential energy as a function of the reaction coordinates for the diffusion-limited two-state enzyme as predicted by MEPP. The enzyme functional states of a free enzyme are (E+S) and (E+P), and (ES) represent an enzyme in complex with a substrate. $S^{‡}$ and $P^{‡}$ are the corresponding transition states. $\Delta G_{1}^{‡}$ and $\Delta G_{2}^{‡}$ are two equal transition-state barriers.

Based on the MEPP, we have also shown that in the most probable thermodynamic state, the maximal optimal reaction flux, which is established for a given thermodynamic force, is limited by diffusion. This result is consistent with the notion of diffusion-limited or catalytically optimal enzymes. The concept of diffusion-limited enzymes is well known, and catalytic optimality in enzyme kinetics is not an idealized theoretical concept. It occurs in nature but is a rare phenomenon. Exemplary enzymes that are supposed to operate close to the optimal catalytic state are the triose phosphate isomerase [4,15,16,41] and a family of β-Lactamase enzymes [11,12,44]. It has been suggested [6,8] that a compromise in the evolution of a simple enzyme function led to the condition where none of the transition-state barriers of a multistep enzyme reaction should be rate-limiting. This means that the activation energies of all reaction steps in an enzyme reaction are equal. In terms of enzyme rate constants, this is equivalent to the globally optimal condition obtained in our analysis based on MEPP ($k_{1\;\mathrm{op}}^{+*}\left[S\right]=k_{2\;\mathrm{op}}^{+}$ and $a_{1\;\mathrm{op}}^{+*}\left[S\right]=a_{2\;\mathrm{op}}^{+}=a_{3\;\mathrm{op}}^{+})$ for the two- and three-state kinetic schemes, respectively. A similar criterion to that predicted by MEPP in this work has been observed in previous kinetic experiments and was used to evaluate the catalytic optimality of β-Lactamase enzymes [44]. Moreover, Heinrich et al. [2] also found in their theoretical studies based on flux maximization that the maximal reaction flux in the two-state kinetic scheme of the enzyme reaction is established under the same condition $k_{1\;}^{+*}\left[S\right]=k_{2}^{+}$ as in our work. A common feature of all these conditions is that catalytically perfect enzymes should not contain any intrinsic rate-limiting reaction steps. Hence, the overall reaction flux in the steady state is limited solely by the diffusion of a substrate to the enzyme’s active site. A diagram of the potential energy as a function of the reaction coordinate for such diffusion-limited or, in other words, catalytically perfect enzyme, as predicted here with MEPP for the two-state scheme, must consist of two equal transition-state barriers, as schematically presented in Figure 5. According to the kinetic scheme presented in Figure 1a) the transition-state barrier of the first reaction step S+E→ES $\left(\Delta G_{1}^{‡}\right)$ is equal to the transition-state barrier of the second reaction step ES→E+P ($\Delta G_{2}^{‡}).$ Applying the well-known Eyring equation, this equality ($\Delta G_{1}^{‡}=\Delta G_{2}^{‡}$) turns out to be $k_{1\;}^{+*}\left[S\right]=k_{2}^{+}$ in terms of kinetic parameters. That is again the same condition as predicted with MEPP in this work.

## 3. Methods and Materials: Theory, General Considerations and Basic Principles

### 3.1. General Considerations

In this work, the irreversible two and three-state kinetic schemes of an enzyme reaction in a non-equilibrium steady state are considered for thermodynamic investigation with MEPP. The corresponding kinetic schemes are presented in Figure 1.

The kinetic schemes shown in Figure 1 should be viewed as simple toy models of an open chemical reactor (e.g., continuous-flow stirred-tank reactor) operating in a non-equilibrium steady state at constant pressure and temperature. The enzyme is immobile (fixed to a solid surface and not carried by the flows) and cannot be destroyed or created within the reactor. The total enzyme concentration in the model is constant, which is considered by the fixed value of the model parameter ${\left[E\right]}_{\mathrm{tot}}$. The solution has an influx $v_{S\;\mathrm{in}}$ with only the substrate molecules as a solute, which yields a known initial substrate concentration within the reactor. As the solution flows through the system, it interacts with the enzyme, and a portion of the substrate molecules is transformed into the product, while the remaining are left over. The solution flowing through the reactor quickly reaches a stationary composition. This is achieved by considering the outgoing fluxes of the leftover substrate molecules $v_{S\;\mathrm{out}}$ and the product molecules $v_{P\;\mathrm{out}}$. The fluxes $v_{S\;\mathrm{in}}$, $v_{S\;\mathrm{out}}$, and $v_{P\;\mathrm{out}}$ are considered constant. A non-equilibrium steady state is established if $v_{S\;\mathrm{in}}=v_{S\;\mathrm{out}}+v_{P\;\mathrm{out}}$. The latter condition, and a fixed total enzyme concentration, imply mass conservation of the reaction system, which is necessary for establishing a non-equilibrium steady state.

Furthermore, it is considered that stationary concentrations [S] and [P] are considerably different from corresponding equilibrium concentrations ${\left[S\right]}_{\mathrm{eq}.}$ and ${\left[P\right]}_{\mathrm{eq}.}$. In this sense, the reactions are irreversible and should not be viewed as thermodynamically ideal reversible processes in (or close to) a thermodynamic equilibrium state. The processes under investigation could not be turned in opposite directions by small changes in reaction parameters [S] and [P]. There is a high overall thermodynamic force (ΔG≪0) of the forward reaction S→P. The latter condition is often fulfilled in experiments where the initial substrate concentration $({\left[S\right]}_{0}$) is much higher than the initial product concentration $({\left[P\right]}_{0}$), i.e., ${\left[S\right]}_{0}\gg {\left[P\right]}_{0}$ (usually ${\left[P\right]}_{0}=0$). The same condition also holds for the in vivo biological reactions, where the product concentration remains low due to continuous product uptake by subsequent reactions [45]. The model setup, in general, follows the conditions discussed in the reference [46].

Our theoretical analysis relies mainly on the principles of statistical thermodynamics. A large number of enzyme molecules is considered by the model parameter ${\left[E\right]}_{\mathrm{tot}}$. Enzyme molecules can be found in different functional enzymatic states, and the occupation of these states is evaluated statistically by the steady-state probabilities $p_{i}$. The Shannon information entropy of the steady-state probability distribution for the occupation of enzyme states is calculated. The time evolution of the concentrations of different enzymatic states is described by the mass-action kinetics, and the optimal enzyme behavior in the steady state is obtained by MEPP. Optimal model solutions are normalized per total enzyme concentration ${\left[E\right]}_{\mathrm{tot}}$. The properties of the system’s statistically most probable steady state are theoretically investigated using thermodynamic optimization principles MEPP, MaxEnt, and other analytical methods such as linear stability analysis and metabolic control analysis (MCA).

### 3.2. Entropy Production

The rate of entropy production in an enzyme reaction is defined as:

(2) $$\sigma =\frac{vX}{T},$$

where v is the overall reaction flux in a steady state, X is the overall thermodynamic force of the reaction or reaction affinity, and T is the absolute temperature, which is considered constant. For both kinetic schemes shown in Figure 1, the overall reaction flux in a steady state is derived by considering the irreversible Haldane–Brigs enzyme model. Detailed modeling for the two-state scheme is given in the next section, “Kinetic and thermodynamic modeling”, and for the three-state scheme in “Appendix A”. The affinity of a reaction is defined as a negative change of the Gibbs free energy (−ΔG) of the step S→P in a steady state:

(3) $$X\equiv -\Delta G=RTln\left(K^{*}\frac{\left[S\right]}{\left[P\right]}\right),$$

where $K^{*}$ is a reaction equilibrium constant, *R* is the universal gas constant, and [S] and [P] are the steady-state substrate and product concentrations, respectively.

According to MEPP, open thermodynamic systems in a steady state are characterized by the maximum entropy production rate. Hence, for both kinetic schemes presented in Figure 1, the rate of entropy production is maximized with respect to the kinetic parameters. According to the basic thermodynamic formulation of MEPP, the maximal rate of entropy production in a steady state is achieved by the maximal reaction flux at a constant thermodynamic force [22,30]. In this case, the elementary rate constants appear as natural variables for flux maximization. Under suitable optimization conditions described later, there is a unique maximum of the reaction flux corresponding to the maximum rate of entropy production according to Equation (2). Optimal solutions for the elementary enzyme rate constants are derived, allowing the derivation of the optimal catalytic and Michaelis–Menten constant, as well as their ratio, known as the enzyme specificity constant.

### 3.3. Shannon Information Entropy

The Shannon information entropy in an enzyme reaction is defined as:

(4) $$H=-\overset{n}{\underset{i=1}{\sum }}p_{i}\ln\left(p_{i}\right),$$

where $p_{i}$ represents the steady-state probability of occupying the *i*-th enzyme functional state, defined as:

(5) $$p_{i}=\frac{\left[x_{i}\right]}{{\left[E\right]}_{\mathrm{tot}}},$$

where [*E*]_tot_ is the total enzyme concentration and $\left[x_{i}\right]$ is the concentration of the *i*-th enzyme state. The Shannon information entropy is expressed as a function of the enzyme rate constants. As in one of our recent works, its maximal value is used as a statistical measure of a reaction system’s most probable steady state [15]. Optimal values of elementary rate constants obtained by maximization of the entropy production rate result in the most uniform probability distribution of enzyme functional states and, thus, in the maximal value of the Shannon information entropy. The Shannon entropy is maximized by considering $\sum_{i}p_{i}=1$.

### 3.4. Sensitivity Analysis

The response or sensitivity of the reaction flux to external signals is analyzed in terms of elasticity coefficients ($\varepsilon_{S}^{v})$ known from Metabolic Control Analysis (MCA). The elasticity coefficient ($\varepsilon_{S}^{v})$ is a quantity that measures the sensitivity of a reaction flux (v) with respect to changes in substrate concentration ([S]) and is defined as [47,48]:

(6) $$\varepsilon_{S}^{v}=\frac{\left(dv/v\right)}{\left(d\left[S\right]/\left[S\right]\right)}=\frac{dln\left(v\right)}{dln\left(\left[S\right]\right)},$$

### 3.5. Stability Analysis

The stability of steady states in enzyme-catalyzed reactions is analyzed by linear stability analysis [36,37]. Each of the kinetic schemes presented in Figure 1 is described by a corresponding kinetic model—a system of ordinary non-linear first-order differential equations of the form:

(7) $$\dot{X}=f\left(X\right),\;X\in {\mathbb{R}}^{n},$$

where **X** is an *n*-dimensional state vector, $\dot{X}$ is its first-order time derivative, and f(X) is a non-linear function. The steady state of Equation (7) is defined as:

(8) $${\dot{X}}_{X=X^{*}}=f\left(X^{*}\right)=0,$$

where $X^{*}$ represents the steady-state solution of Equation (7). The stability of this steady state is investigated by the linearization of f(X) in the vicinity of $X^{*}$. For this purpose, the Jacobian matrix (J) of the system is constructed, and the trace of the Jacobian matrix (Tr(J)) is calculated. According to references [36,37,38,39,40], stable and rigid steady states are characterized by Tr(J)≪0, while stable and flexible steady states are characterized by negative values of Tr(J) slightly smaller than zero. We investigate how Tr(J) behaves under maximal entropy production rate and maximal Shannon information entropy. Hence, we investigate the stability of an enzyme reaction’s most likely steady state.

### 3.6. Optimization Conditions

In our previous works [13,14,15], we investigated the conditions for the existence of a unique maximum in the rate of entropy production for two- and four-state reversible enzyme kinetic schemes. We have proposed a general condition that can be used to analyze reversible and irreversible kinetic schemes [13,14,15]. The same condition is also used in this work. In short, the basic constraint considered is that the overall change in Gibbs free energy within the transformation of one substrate molecule into a product depends solely on the chemical nature of the substrate and the product and not on the kinetic properties of the enzyme [2,34,41]. This well-known and widely accepted constraint implies that the overall equilibrium constant $\left(K^{*}\right)$ is a fixed reaction parameter. The equilibrium constant ($K^{*}$) can be further expressed in terms of forward and backward enzyme rate constants $(k_{i}^{\pm }$) as:

(9) $$K^{*}=\frac{k_{1}^{+*}k_{2}^{+}k_{3}^{+}\ldots k_{n}^{+}}{k_{1}^{-}k_{2}^{-}k_{3}^{-}\ldots k_{n}^{-*}}.$$

It follows from Equation (9) that enzyme rate constants cannot be varied independently. Variation of any arbitrarily chosen rate constant should come across with the variation of one or more of the other rate constants so that $K^{*}$ remains unchanged. The equilibrium constant K* represents the intrinsic relationship between enzyme rate constants under arbitrary conditions, either within the equilibrium, the steady state, or the time-dependent transient state [34]. However, as shown in [13], the constraint of a fixed K* does not directly imply the existence of a unique maximum of the entropy production rate. It applies to physical situations where the entropy production rate asymptotically approaches a finite and maximum value for infinite values of the enzyme rate constant. It is known that the values of the enzyme rate constants $(k_{i}^{\pm }$) cannot be infinite and are limited upwards by several factors [2,3,49,50,51,52,53,54,55]. In agreement with this finding, we explicitly require that a unique maximum in the rate of entropy production occurs for non-infinite values of the rate constants, which is reached when the products $K^{+}$ and $K^{-}$ of all forward and backward enzyme rate constants, respectively, are also fixed parameters [13,14,15]:

(10) $$K^{+}=k_{1}^{+*}k_{2}^{+}k_{3}^{+}\ldots k_{n}^{+},$$

(11) $$K^{-}=k_{1}^{-}k_{2}^{-}k_{3}^{-}\ldots k_{n}^{-*}.$$

The constraints given in Equations (10) and (11) are consistent with Equation (9). Since, in this work, irreversible kinetic schemes are investigated, the constraint given in Equation (10) is of particular importance. Under this constraint, the entropy production rate, Shannon information entropy, and the trace of the Jacobian matrix are maximized with respect to enzyme rate constants. Such a state of the system can be calculated using standard mathematical procedures for finding extreme values of functions of one or more variables.

### 3.7. Kinetic and Thermodynamic Modeling of the Irreversible Two-State Kinetic Scheme

The kinetic scheme of the irreversible two-state enzyme reaction is shown in Figure 1a. The system variables are concentrations of substrate [S], product [P], free enzyme [E], and enzyme in complex with the substrate [ES]. The time evolution of these variables is determined with the kinetic model that represents a dynamical system of four ordinary differential equations:

(12) $$\frac{d\left[S\right]}{dt}=v_{S\;\mathrm{in}}-k_{1}^{+*}\left[S\right]\left[E\right]-v_{S\;\mathrm{out}},$$

(13) $$\frac{d\left[P\right]}{dt}=k_{2}^{+}\left[ES\right]-v_{P\;\mathrm{out}},$$

(14) $$\frac{d\left[E\right]}{dt}=-k_{1}^{+*}\left[S\right]\left[E\right]+k_{2}^{+}\left[ES\right],$$

(15) $$\frac{d\left[ES\right]}{dt}=k_{1}^{+*}\left[S\right]\left[E\right]-k_{2}^{+}\left[ES\right],$$

where $k_{i}^{+}$ are the enzyme forward rate constants, while $v_{S\;\mathrm{in}}$, $v_{S\;\mathrm{out}}$, and $v_{P\;\mathrm{out}}$ are the external metabolic fluxes, as indicated in Figure 1a. In the model, it is assumed that enzyme molecules are not carried in or out of the reaction system by external fluxes. Thus, by summing up Equations (14) and (15), it follows that d/dt ([E]+[ES])=0, which means that the total enzyme concentration (${\left[E\right]}_{\mathrm{tot}})$ is a conserved quantity:

(16) $${\left[E\right]}_{\mathrm{tot}}=\left[E\right]+\left[ES\right].$$

By considering $v_{S\;\mathrm{in}}=v_{S\;\mathrm{out}}+v_{P\;\mathrm{out}}$ and summing up Equations (12), (13) and (15) yield d/dt ([S]+[ES]+[P])=0, which implies that the sum [S](t)+[ES](t)+[P](t) is constant at any time and is, hence, a conserved quantity. The latter condition and the constant total enzyme concentration (Equation (16)) imply the conservation of the mass of the entire reaction system ending up in an actual non-equilibrium steady state.

Considering Equations (14) and (16) under steady-state conditions, we obtain the system of two algebraic equations with steady-state concentrations [E] and [ES] as unknown quantities:

(17) $$k_{1}^{+*}\left[S\right]\left[E\right]-k_{2}^{+}\left[ES\right]=0,$$

(18) $${\left[E\right]}_{\mathrm{tot}}=\left[E\right]+\left[ES\right],$$

with the corresponding steady-state solutions:

(19) $$\left[E\right]={\left[E\right]}_{\mathrm{tot}}\frac{k_{2}^{+}}{k_{2}^{+}+k_{1}^{+*}\left[S\right]},$$

(20) $$\left[ES\right]={\left[E\right]}_{\mathrm{tot}}\frac{k_{1}^{+*}\left[S\right]}{k_{2}^{+}+k_{1}^{+*}\left[S\right]}.$$

Considering that the velocity of the product formation for the reaction presented in Figure 1a is $v=k_{2}^{+}\left[ES\right]$, and that the steady-state concentration [ES] is given with Equation (20), the overall steady-state reaction flux is expressed as:

(21) $$v=\frac{{\left[E\right]}_{\mathrm{tot}}\;k_{2}^{+}k_{1}^{+*}\left[S\right]}{k_{2}^{+}+k_{1}^{+*}\left[S\right]}.$$

The above expression can be rearranged into a standard form:

(22) $$v=\frac{v_{\max}\;\left[S\right]}{K_{M}+\left[S\right]},$$

where $v_{\max}=k_{\mathrm{cat}}{\left[E\right]}_{\mathrm{tot}}$ is the saturated reaction flux achieved for [S]→∞, and $K_{M}$ is a half saturation constant known as the Michaelis–Menten constant. Here, the reaction parameters $k_{\mathrm{cat}}$ and $K_{M}$ are:

(23) $$k_{\mathrm{cat}}=k_{2}^{+},$$

(24) $$K_{M}=\frac{k_{2}^{+}}{k_{1}^{+*}}.$$

By considering Equations (4), (5), (19) and (20), the Shannon information entropy expressed as a function of the enzyme rate constants reads:

(25) $$H=-\frac{k_{2}^{+}}{k_{2}^{+}+k_{1}^{+*}\left[S\right]}\ln\left(\frac{k_{2}^{+}}{k_{2}^{+}+k_{1}^{+*}\left[S\right]}\right)-\frac{k_{1}^{+*}\left[S\right]}{k_{2}^{+}+k_{1}^{+*}\left[S\right]}\ln\left(\frac{k_{1}^{+*}\left[S\right]}{k_{2}^{+}+k_{1}^{+*}\left[S\right]}\right).$$

The trace of the Jacobian of the system of Equations (12)–(15) is:

(26) $$Tr\left(J\right)=Tr\left(\begin{array}{cccc} -k_{1}^{+*}\left[E\right] & 0 & -k_{1}^{+*}\left[S\right] & 0\\ 0 & 0 & 0 & k_{2}^{+}\\ -k_{1}^{+*}\left[E\right] & 0 & -k_{1}^{+*}\left[S\right] & k_{2}^{+}\\ k_{1}^{+*} & 0 & k_{1}^{+*}\left[S\right] & -k_{2}^{+} \end{array}\right)=-\left(k_{1}^{+*}\left[S\right]+k_{2}^{+}\right),$$

In the derivation of Equation (26), we considered a much lower concentration of the enzyme than that of the substrate ([E]≪[S]).

If we take the natural logarithm of Equation (22) and then calculate the first derivative of the resulting expression with respect to [S], the elasticity coefficient ($\varepsilon_{S}^{v}$), defined with Equation (6), is:

(27) $$\varepsilon_{S}^{v}=\frac{K_{M}}{K_{M}+\left[S\right]}.$$

The reaction velocity Equation (21), the Shannon information entropy Equation (25), and the trace of the Jacobian matrix Equation (26) are then all optimized with respect to enzyme rate constants by considering the optimization condition Equation (10). For the irreversible two-state kinetic scheme, this condition is $K^{+}=k_{1}^{+*}k_{2}^{+}$. According to this condition, the rate constants $k_{1}^{+*}$ and $k_{2}^{+}$ could not be considered independent variables simultaneously. If, for example, $k_{2}^{+}$ is taken as an independent variable, $k_{1}^{+*}$ must vary according to Equation (10): $k_{1}^{+*}=K^{+}/k_{2}^{+}$. Therefore, if in Equations (21), (25) and (26), $k_{1}^{+*}$ is replaced by the ratio $K^{+}/k_{2}^{+}$, then all quantities v, H, and Tr(J) become functions of a single variable $k_{2}^{+}$:

(28) $$v\left(k_{2}^{+}\right)=\frac{{\left[E\right]}_{\mathrm{tot}}\;K^{+}\left[S\right]}{k_{2}^{+}+\frac{K^{+}}{k_{2}^{+}}\left[S\right]},$$

(29) $$H\left(k_{2}^{+}\right)=-\frac{k_{2}^{+}}{k_{2}^{+}+\frac{K^{+}}{k_{2}^{+}}\left[S\right]}\ln\left(\frac{k_{2}^{+}}{k_{2}^{+}+\frac{K^{+}}{k_{2}^{+}}\left[S\right]}\right)-\frac{\frac{K^{+}}{k_{2}^{+}}\left[S\right]}{k_{2}^{+}+\frac{K^{+}}{k_{2}^{+}}\left[S\right]}\ln\left(\frac{\frac{K^{+}}{k_{2}^{+}}\left[S\right]}{k_{2}^{+}+\frac{K^{+}}{k_{2}^{+}}\left[S\right]}\right),$$

(30) $$Tr\left(J\right)\left(k_{2}^{+}\right)=-\left(k_{2}^{+}+\frac{K^{+}}{k_{2}^{+}}\left[S\right]\right).$$

Equations (28)–(30) are essential in optimizing the two-state irreversible kinetic scheme. They could also be rewritten for the case of taking the rate constant $k_{1}^{+*}$ as an independent variable. The choice of variables ($k_{2}^{+}$ or $k_{1}^{+*}$) does not affect the final optimization solutions.

In Section 2. Results and Discussion, we first describe the optimization of the two-state kinetic scheme shown in Figure 1a. We then compare all optimal solutions for the two- and three-state irreversible kinetic schemes. All derivations and calculations for the three-state kinetic scheme are presented in Appendix A to avoid repetitions of similar expressions.

## 4. Conclusions

The theoretical analysis of chemical kinetic mechanisms in the context of self-organization can contribute significantly to a better understanding of the organization and evolution of biological systems. Such mechanisms are closely related to the fluxes of matter required in open systems to absorb, generate, and export entropy. According to the second law of thermodynamics, open systems with effective mechanisms responsible for exporting internally generated entropy to the environment can organize themselves much better, avoid the accumulation of entropy within the system, and thus survive in non-equilibrium (steady) states that are characteristic of biological systems. In this context, we have studied the linear kinetic mechanisms of enzymatic reactions with MEPP. The influence of the kinetic mechanism on the ability of the enzyme-catalyzed reaction to self-organize is examined. Several thermodynamic quantities and kinetic parameters are analyzed to quantitatively evaluate these aspects.

A widely accepted view is that ancestral enzymes were generalists, catalyzing a broad set of substrates from which modern highly specialized enzymes evolved [35,56,57]. Ancestral enzymes exhibited broad substrate specificity due to complex structures with multiple catalytic sites [35,56] and were also relatively slow catalysts [4,56]. It is proposed [35,56] that evolutionary selection pressure towards increased catalytic activity may have resulted in more specialized enzymes with higher enzyme specificity. Such a long-term process of enzymatic evolutionary specialization might have resulted in a loss of catalytic activity for other possible substrates [56,58]. This means some enzymatic functional states could have considerably changed their stability during specialization or even vanished. If such molecular events have occurred during evolution, this may have affected the number of representative enzyme functional states and, thus, also the kinetic mechanisms of enzyme reactions. From this point of view, it seems natural to test how the essential reaction parameters in enzyme-catalyzed reactions depend on kinetic reaction mechanisms. Our theoretical analysis indicates that optimal enzyme performance depends on the number of enzyme functional states or elementary reaction steps. Based on the MEPP, which is assumed here to be an essential criterion for enzyme evolution, it is found that kinetic mechanisms with a lower number of enzymatic states may result in higher entropy production rate, which, according to widely accepted view [35,56], might be a characteristic of higher-evolved specialized enzymes (so-called enzyme specialists). Our results show considerable intrinsic differences between two- and three-state kinetic mechanisms with a clear indication that entropy decreases due to a loss of a single internal enzymatic functional state resulting in increased entropy production rate and increased enzyme catalytic activity and higher enzyme sensitivity to external perturbations. In addition, the basic enzymatic kinetic parameters such as Michaelis–Menten and specificity constants are also considerably higher for the two-state kinetic scheme than the three-state scheme under the same conditions. Theoretical results presented in this work indicate that loss of stability in functional enzymatic states or their complete extinction might have been critical molecular events in the evolution of fast specialized enzymes. Within a system consisting of a large number of molecules, this results in a loss of a high number of degrees of freedom within the system and increases the export of entropy from the system to its environment. As a consequence, the entire macroscopic system may become better internally organized.

## Figures and Tables

**Table 1:** Comparison of the parameters accounting for optimal enzyme performance within two- and three-state kinetic schemes. Calculations for both kinetic schemes are performed under identical conditions: [S]=1 μM, $K^{+}=100\;{\mu M}^{-1}s^{-2}$ (for the two-state kinetic scheme) and [S]=1 μM, $K^{+}=100\;{\mu M}^{-1}s^{-3}$ (for the three-state kinetic scheme) within the most probable steady state characterized by the maximal Shannon information entropy, maximal rate of entropy production, and maximal trace of the Jacobian matrix.

Quantity and Its Unit	Two-State Kinetic Scheme		Three-State Kinetic Scheme	
	Expression	Value	Expression	Value
$k_{\;\mathrm{op}}^{+},\;a_{\;\mathrm{op}}^{+}\;\left[s^{-1}\right]$	$k_{\mathrm{op}}^{+}=\sqrt{K^{+}\left[S\right]}$	$10\;s^{-1}$	$a_{\mathrm{op}}^{+}=\sqrt[{3}]{K^{+}\left[S\right]}$	$4.6\;s^{-1}$
$v\;/{\left[E\right]}_{\mathrm{tot}}\;\left[{\mu \mathrm{Ms}}^{-1}\right]$	$k_{\;\mathrm{op}}^{+}/2$	$5\;s^{-1}$	$a_{\;\mathrm{op}}^{+}/3$	$1.5\;s^{-1}$
H [/]	Equation (29)	ln(2)	Equation (A14)	ln(3)
$Tr\left(J\right)\;\left[s^{-1}\right]$	$-2k_{\;\mathrm{op}}^{+}$	$-20{\;s}^{-1}$	$-3a_{\;\mathrm{op}}^{+}$	$-13.8{\;s}^{-1}$
$k_{\mathrm{cat}},\;a_{\mathrm{cat}}\;\left[s^{-1}\right]$	$k_{\;\mathrm{op}}^{+}$	$10\;s^{-1}$	$a_{\;\mathrm{op}}^{+}/2$	$2.3\;s^{-1}$
$K_{M},\;A_{M}\;\left[\mu M\right]$	$K_{M}=\left[S\right]$	1.0 μM	$A_{M}=\left[S\right]/2$	0.5 μM
$\left(\frac{k_{\mathrm{cat}}}{K_{M}}\right),\;\left(\frac{a_{\mathrm{cat}}}{A_{M}}\right)\;\left[{\left(\mu \mathrm{Ms}\right)}^{-1}\right]$	$\frac{k_{\mathrm{cat}}}{K_{M}}=k_{\;1\;\mathrm{op}}^{+*}$	$10\;{\left(\mu \mathrm{Ms}\right)}^{-1}$	$\frac{a_{\mathrm{cat}}}{A_{M}}=a_{\;1\;\mathrm{op}}^{+*}$	$4.6\;{\left(\mu \mathrm{Ms}\right)}^{-1}$
$\varepsilon_{S}^{v}\;\left[/\right]$	Equation (27)	1/2	Equation (27)	1/3

## Data Availability

No new data were created or analysed in this study. Data Sharing is not applicable to this article.

## Appendix A. Mathematical Modeling of the Three-State Kinetic Scheme

The kinetic scheme of the irreversible three-state enzyme reaction is shown in the main text in Figure 1b. In this case, the system variables are the concentrations of substrate [S], product [P], free enzyme [E], the enzyme in a complex with the substrate [ES], and the enzyme in complex with the product [EP]. The time evolution of these variables is determined with the following dynamical system:

(A1) $$\frac{d\left[S\right]}{dt}=v_{S\;\mathrm{in}}-a_{1}^{+*}\left[S\right]\left[E\right]-v_{S\;\mathrm{out}},$$

(A2) $$\frac{d\left[P\right]}{dt}=a_{3}^{+}\left[EP\right]-v_{P\;\mathrm{out}},$$

(A3) $$\frac{d\left[E\right]}{dt}=-a_{1}^{+*}\left[S\right]\left[E\right]+a_{3}^{+}\left[EP\right],$$

(A4) $$\frac{d\left[ES\right]}{dt}=a_{1}^{+*}\left[S\right]\left[E\right]-a_{2}^{+}\left[ES\right],$$

(A5) $$\frac{d\left[EP\right]}{dt}=a_{2}^{+}\left[ES\right]-a_{3}^{+}\left[EP\right],$$

where $a_{i}^{+}$ are the enzyme forward rate constants. As in the two-state kinetic scheme, the summation of Equations (A3)–(A5) gives the conservation of the total enzyme concentration:

(A6) $${\left[E\right]}_{\mathrm{tot}}=\left[E\right]+\left[ES\right]+\left[EP\right].$$

Summation of Equations (A1), (A2), (A4) and (A5) under the condition $v_{S\;\mathrm{in}}=v_{S\;\mathrm{out}}+v_{P\;\mathrm{out}}$ yields [S](t)+[ES](t)+[EP](t)+[P](t) being a conserved quantity. Thus, the mass of the entire system is constant, and the reaction is in a non-equilibrium steady state. By considering Equations (A3), (A4) and (A6) under steady-state conditions, we obtain the system of three algebraic equations with steady-state concentrations [E], [ES], and [EP] as unknown quantities:

(A7) $$-a_{1}^{+*}\left[S\right]\left[E\right]+a_{3}^{+}\left[ES\right]=0,$$

(A8) $$a_{1}^{+*}\left[S\right]\left[E\right]-a_{2}^{+}\left[ES\right]=0,$$

(A9) $${\left[E\right]}_{\mathrm{tot}}=\left[E\right]+\left[ES\right]+\left[EP\right].$$

The corresponding steady-state solutions are:

(A10) $$\left[E\right]={\left[E\right]}_{\mathrm{tot}}\frac{a_{2}^{+}a_{3}^{+}}{a_{1}^{+*}\left[S\right]a_{2}^{+}+a_{1}^{+*}\left[S\right]a_{3}^{+}+a_{2}^{+}a_{3}^{+}},$$

(A11) $$\left[ES\right]={\left[E\right]}_{\mathrm{tot}}\frac{a_{1}^{+*}\left[S\right]a_{3}^{+}}{a_{1}^{+*}\left[S\right]a_{2}^{+}+a_{1}^{+*}\left[S\right]a_{3}^{+}+a_{2}^{+}a_{3}^{+}},$$

(A12) $$\left[EP\right]={\left[E\right]}_{\mathrm{tot}}\frac{a_{1}^{+*}\left[S\right]a_{2}^{+}}{a_{1}^{+*}\left[S\right]a_{2}^{+}+a_{1}^{+*}\left[S\right]a_{3}^{+}+a_{2}^{+}a_{3}^{+}}.$$

These steady-state concentrations (Equations (A10)–(A12)) are later used to derive the steady-state reaction flux and the Shannon information entropy for that case.

The optimization condition (10) (see the main text) for the three-state kinetic scheme is $K^{+}=a_{1}^{+*}a_{2}^{+}a_{3}^{+}$. In this case, there are three variables. Two can be chosen as independent, while the third must vary so that the product $K^{+}$ remains fixed. There are three ways to determine two independent and one dependent variable. First, $a_{1}^{+*}$ and $a_{2}^{+}$ could be independent and $a_{3}^{+}$ the dependent variable that varies according to Equation (10), i.e., $a_{3}^{+}=K^{+}/\left(a_{1}^{+*}a_{2}^{+}\right)$. If $a_{1}^{+*}$ and $a_{3}^{+}$ are independent, then $a_{2}^{+}=K^{+}/\left(a_{1}^{+*}a_{3}^{+}\right)$, and, if $a_{2}^{+}$ and $a_{3}^{+}$ are independent, then $a_{1}^{+*}=K^{+}/\left(a_{2}^{+}a_{3}^{+}\right)$. As explained in the main text, optimization results are always the same and do not depend on the choice of independent variables. Here we chose $a_{2}^{+}$ and $a_{3}^{+}$ as independent variables and consider $a_{1}^{+*}=K^{+}/\left(a_{2}^{+}a_{3}^{+}\right)$ in Equations (A10)–(A12).

The rate of product formation for that case is $v=a_{3}^{+}\left[EP\right]$. Hence, by considering Equation (A12) for [EP] and $a_{1}^{+*}=K^{+}/\left(a_{2}^{+}a_{3}^{+}\right)$ in the latter equation, the overall steady-state reaction flux for the three-state kinetic scheme presented in Figure 1b) is:

(A13) $$v\left(a_{2}^{+},a_{3}^{+}\right)=\frac{{\left[E\right]}_{\mathrm{tot}}K^{+}\;\left[S\right]}{a_{2}^{+}a_{3}^{+}+\frac{K^{+}\left[S\right]}{a_{2}^{+}}+\frac{K^{+}\left[S\right]}{a_{3}^{+}}}.$$

Considering Equations (4), (5) and (A10)–(A12), and $a_{1}^{+*}=K^{+}/\left(a_{2}^{+}a_{3}^{+}\right)$ the Shannon information entropy for that case is:

(A14) $$\begin{array}{cc} H\left(a_{2}^{+},a_{3}^{+}\right)= & -\frac{a_{2}^{+}a_{3}^{+}}{a_{2}^{+}a_{3}^{+}+\frac{K^{+}\left[S\right]}{a_{2}^{+}}+\frac{K^{+}\left[S\right]}{a_{3}^{+}}}ln\left(\frac{a_{2}^{+}a_{3}^{+}}{a_{2}^{+}a_{3}^{+}+\frac{K^{+}\left[S\right]}{a_{2}^{+}}+\frac{K^{+}\left[S\right]}{a_{3}^{+}}}\right)\\ & \;-\frac{\frac{K^{+}\left[S\right]}{a_{2}^{+}}}{a_{2}^{+}a_{3}^{+}+\frac{K^{+}\left[S\right]}{a_{2}^{+}}+\frac{K^{+}\left[S\right]}{a_{3}^{+}}}ln\left(\frac{\frac{K^{+}\left[S\right]}{a_{2}^{+}}}{a_{2}^{+}a_{3}^{+}+\frac{K^{+}\left[S\right]}{a_{2}^{+}}+\frac{K^{+}\left[S\right]}{a_{3}^{+}}}\right)\\ & \;-\frac{\frac{K^{+}\left[S\right]}{a_{3}^{+}}}{a_{2}^{+}a_{3}^{+}+\frac{K^{+}\left[S\right]}{a_{2}^{+}}+\frac{K^{+}\left[S\right]}{a_{3}^{+}}}ln\left(\frac{\frac{K^{+}\left[S\right]}{a_{3}^{+}}}{a_{2}^{+}a_{3}^{+}+\frac{K^{+}\left[S\right]}{a_{2}^{+}}+\frac{K^{+}\left[S\right]}{a_{3}^{+}}}\right). \end{array}$$

By considering the system of Equations (A1)–(A5), the condition [E]≪[S] and $a_{1}^{+*}=K^{+}/\left(a_{2}^{+}a_{3}^{+}\right)$, the trace of the Jacobian matrix is:

(A15) $$Tr\left(J\right)=-\left(\frac{K^{+}}{a_{2}^{+}a_{3}^{+}}\left[S\right]+a_{2}^{+}+a_{3}^{+}\right).$$

The overall steady-state reaction flux can be expressed in the form of Equation (22) with the catalytic constant ($a_{\mathrm{cat}}$) and the Michaelis–Menten constant ($A_{M}$) defined as:

(A16) $$a_{\mathrm{cat}}=\frac{a_{2}^{+}a_{3}^{+}}{a_{2}^{+}+a_{3}^{+}},$$

(A17) $$A_{M}=\frac{a_{2}^{+}a_{3}^{+}}{a_{1}^{+*}(a_{2}^{+}+a_{3}^{+})}.$$

The elasticity coefficient $\varepsilon_{S}^{v}$ is the same as for the two-state irreversible kinetic scheme (Equation (27)).

The central result obtained by maximization of Equation (A13) is the optimality condition in the form $a_{\mathrm{op}}^{+}=a_{1\;\mathrm{op}}^{+*}\left[S\right]=a_{2\;\mathrm{op}}^{+}=a_{3\;\mathrm{op}}^{+}$ with $a_{\mathrm{op}}^{+}=\sqrt[{3}]{K^{+}\left[S\right]}$ that accounts for the maximal entropy production rate. Hence, the maximal reaction flux normalized by the total enzyme concentration predicted by MEP is $v/{\left[E\right]}_{\mathrm{tot}}=a_{\;\mathrm{op}}^{+}/3$. As in the two-state kinetic scheme, the insertion of $a_{\mathrm{op}}^{+}=a_{1\;\mathrm{op}}^{+*}\left[S\right]=a_{2\;\mathrm{op}}^{+}=a_{3\;\mathrm{op}}^{+}$ into Equations (A10)–(A12) gives equal steady-state concentrations $\left[E\right]=\left[ES\right]=\left[EP\right]={\left[E\right]}_{\mathrm{tot}}/3$. The definition $p_{i}=\left[x_{i}\right]/{\left[E\right]}_{\mathrm{tot}}$ yields a uniform steady-state probability distribution of an enzyme’s functional states $p_{1}=p_{2}=p_{3}=1/3$. Therefore, according to Equation (A14), maximal Shannon information entropy is H=ln(3). For the same optimality condition, the trace of the Jacobian matrix (Equation (A15)) achieves its maximal (least negative) value $\mathrm{Tr}\left(J\right)=-3a_{\mathrm{op}}^{+}$, the optimal catalytic constant ($a_{\mathrm{cat}})$ (Equation (A16)) is $a_{\mathrm{cat}}=a_{\mathrm{op}}^{+}/2$, and the optimal Michaelis–Menten constant (Equation (A17)) is $A_{M}=\left[S\right]/2$. Hence, the optimal specificity constant is $\left(a_{\mathrm{cat}}/A_{M}\right)=a_{1\;\mathrm{op}}^{+*}$. Since it has the same magnitude as the second-order diffusion rate constant, that is a sign of a diffusion-limited and catalytically perfect enzyme.
